# Knowledge and Practices Regarding Dietary Supplements Among Healthcare Professionals in Poland

**DOI:** 10.3390/nu16213691

**Published:** 2024-10-29

**Authors:** Justyna Strocka, Urszula Religioni, Katarzyna Plagens-Rotman, Agnieszka Drab, Piotr Merks, Justyna Kaźmierczak, Eliza Blicharska, Jarosław Pinkas

**Affiliations:** 1 School of Public Health, Centre of Postgraduate Medical Education of Warsaw, 01-826 Warsaw, Poland; justyna_strocka@onet.pl (J.S.);; 2 Center for Sexology and Pediatric, Adolescent Gynecology, Division of Gynecology, Department of Perinatology and Gynecology, Poznan University of Medical Sciences, 60-535 Poznan, Poland; plagens.rotman@gmail.com; 3 Department of Medical Informatics and Statistics with e-Health Laboratory, Medical University of Lublin, 20-093 Lublin, Poland; 4 Department of Pharmacology and Clinical Pharmacology, Faculty of Medicine, Collegium Medicum, Cardinal Stefan Wyszyński University, 01-815 Warsaw, Poland; 5 The Polish Pharmacy Practice Research Network (PPPRN), 02-097 Warsaw, Poland; 6 Department of Analytical Chemistry, Medical University of Lublin, 20-093 Lublin, Poland

**Keywords:** dietary supplements, healthcare professionals, medical practice, quality of care

## Abstract

Introduction: The growing popularity of dietary supplements in Poland raises the need to verify the knowledge and practices of healthcare workers who have a key role in recommending their use. Understanding these issues is important to ensure patient safety and the correct use of dietary supplements. Therefore, the aim of the study was to examine the level of knowledge, practices, and sources of information on dietary supplements among various professional groups of healthcare workers. Material and Methods: The study conducted from September 2023 to June 2024 involved 743 healthcare professionals, including doctors, pharmacists, and nurses. Data were collected online using an original questionnaire. The questionnaire was placed in portals associated with target groups. Results: A total of 73.49% of respondents declared knowledge of dietary supplements. The most common source of information was scientific articles (42.8%), and only 7.00% of respondents used official sources such as the Chief Sanitary Inspectorate. Advertisements had a smaller impact on doctors (*p* < 0.001), with 46.97% of respondents stating that they did not pay attention to them. In addition, 84.52% of respondents considered the composition of the dietary supplement to be a key factor in their recommendation. Conclusions: Knowledge of dietary supplements among healthcare professionals varies, particularly between different professional groups. The results indicate a need for further education and standardization of information to ensure safe and responsible recommendation of supplements to consumers.

## 1. Introduction

The global dietary supplement market has experienced significant growth, with an increasing number of people turning to supplements as a means of improving their health and well-being. This trend is not limited to any one region; studies have shown that in many parts of the world, including Europe, North America, and Asia, the consumption of dietary supplements is becoming more widespread. Factors contributing to this global rise include heightened health awareness, the desire to prevent chronic diseases, and the increasing accessibility of dietary supplements without a prescription. Furthermore, the COVID-19 pandemic has further accelerated this trend as consumers sought ways to boost their immunity and protect against infections, leading to a substantial increase in the sales and consumption of dietary supplements worldwide [1,2].

The growing availability, diversity of preparations, and extensive marketing campaigns have made supplementation a popular element of health prevention as well as a tool supporting the daily diet. Consumers reach for dietary supplements for various reasons: to improve their general health, increase physical fitness, improve well-being, and also to counteract the effects of aging [1,3]. This phenomenon, referred to as the “supplement use phenomenon” [4], gained particular intensity during the COVID-19 pandemic when sales of dietary supplements in many countries increased by up to 50% [5]. In Poland, between 2017 and 2020, the Chief Sanitary Inspectorate (GIS, pol. Główny Inspektorat Sanitarny) received almost 63,000 notifications about the introduction of new products to the market [6], which further emphasizes the dynamic development of this segment.

The consumption of dietary supplements has grown significantly both in Poland and globally. In Poland, about 10% of adults regularly use dietary supplements, with vitamins B6, C, and D being the most commonly consumed supplement, followed by magnesium and zinc. Studies conducted across various European countries have demonstrated a wide variation in food supplement consumption levels, ranging from 8% to 60% [7].

With the growing popularity of dietary supplements, more questions are being raised about their safety, effectiveness, and appropriate use. Given the often contradictory information available, the role of healthcare professionals, such as doctors, pharmacists, nurses, and dietitians, becomes crucial. While pharmacists frequently provide advice on supplementation, it is essential to recognize that other healthcare workers also play an important role in guiding patients and offering reliable information in this area [8,9].

Despite the key role played by healthcare professionals in advising consumers, their knowledge about dietary supplements is often heterogeneous and not always supported by scientific research [10]. The lack of coherent educational programs or differences in legal regulations mean that specialists often encounter difficulties in providing credible recommendations that are consistent with current knowledge. In light of these challenges, research into the knowledge and practices of healthcare workers regarding dietary supplements becomes essential.

Taking the above into account, the main objective of this article is to assess the knowledge and practices of healthcare workers regarding dietary supplements. The study aims to identify the main sources of information about the dietary supplements that specialists draw from, as well as to assess their perception of the effectiveness and safety of these preparations. The study also aimed to understand the extent to which healthcare workers are able to distinguish dietary supplements from medicinal preparations and what approach they take in providing recommendations to consumers. The novelty of this study lies in its comprehensive evaluation of healthcare professionals from various fields—physicians, pharmacists, nurses, and pharmacy technicians—offering a comparative perspective across different roles in the healthcare system. Additionally, this research provides contemporary data, capturing post-pandemic trends in dietary supplement use and how the COVID-19 pandemic has influenced healthcare workers’ practices and perceptions regarding dietary supplements. By investigating both the sources of information and the ability to distinguish dietary supplements from medicinal products, the study provides unique insights into the current state of healthcare professionals’ knowledge in this rapidly evolving field.

## 2. Material and Methods

### 2.1. Study Design and Statistical Analysis

The study was conducted on the basis of proprietary questionnaires of a survey nature. Data were collected online using portals associating target groups in the period September 2023–June 2024. The survey was addressed to healthcare workers: doctors, pharmacists and pharmacy technicians, as well as nurses and midwives.

Consent to the study was issued by the Karol Marcinkowski University of Medical Sciences in Poznań (KB 284/23).

The questionnaire was divided into two parts. The metrics (six questions) included information on gender, age, place of residence, profession, and length of service. Respondents also indicated the place where they currently work.

The second part of the questionnaire (ten questions) contained the relevant questions relating to dietary supplements. First, respondents were asked whether they were declaratively familiar with the concept of a dietary supplement. The assessment was based on a 5-point Likert scale. Healthcare workers were also asked where they obtained knowledge about this category of preparations, where to purchase dietary supplements, and the frequency of recommendations based on a previously prepared list. The remaining questions on a 5-point Likert scale had to do with the differences between dietary supplements and medicinal preparations, safety, quality and applicable regulations to which dietary supplements are subject.

The questionnaire ended with additional, open-ended questions. Respondents were asked about situations in which dietary supplements were recommended, the interview conducted before the recommendation, and the type of supplements that consumers most often choose.

Prior to the full study, a pilot test was conducted with six healthcare workers (three pharmacists, two doctors, and one nurse) to assess the clarity and appropriateness of the questionnaire. None of the participants suggested significant changes, and the questionnaire was deemed suitable for use.

The statistical analyses have been performed using the statistical suite StatSoft. Inc., Tulsa, OK, USA (2017). STATISTICA (data analysis software system) version 13.0. www.statsoft.com.

The quantitive variables were characterized by the arithmetic mean of standard deviation or median (1st–3rd quartile) or max/min (range) and 95% confidence interval. The qualitative variables were presented using count and percentage.

In order to check if a quantitive variable derives from a population of normal distribution, the W Shapiro–Wilk test was used. To determine dependence, strength, and direction between variables, Pearson’s chi-square test, Cramer’s V, and the Mann-Whitney U test were used. In all the calculations, the statistical significance level of *p* = 0.05 was used.

### 2.2. Sample

The study included 743 respondents, of whom more than half (53.84%) were women, with a median age of 44 years (range: 36–51 years). The youngest respondent was 22 years old, while the oldest was 70. Over half of the respondents were doctors (54.24%), and nearly one in four (28.13%) were pharmacists or pharmacy technicians. The median number of years in the profession was 15 years (range: 9–22 years), with the shortest work experience being one year and the longest 53 years. About one-third of the respondents held a specialist title (32.18%). Slightly less than half of the respondents lived in a city with more than 500,000 inhabitants (39.17%), with the majority of them coming from the Masovian Voivodeship (Table 1).

## 3. Results

**Figure 1:**
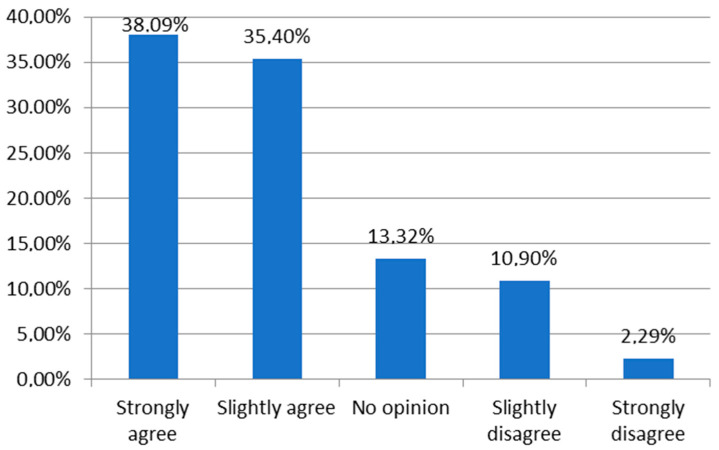
To what extent do you agree with the statement “I understand and know what a dietary supplement is”.

The vast majority of respondents strongly agreed (38.09%) or slightly agreed (35.40%) with the statement, “I understand and know what a dietary supplement is”. Women (*p* < 0.001), pharmacists/pharmacy technicians (*p* < 0.001), and those with a specialist title (*p* < 0.001) were more likely to agree with this statement (Figure 1).

The most common source of knowledge about dietary supplements for respondents was professional articles (42.80%), while the least common was the website of the Chief Sanitary Inspectorate (7.00%). Television, the Internet, and industry conferences were used almost equally by respondents (about one-third in each case).

Men more frequently obtained knowledge from television (*p* = 0.03), medical representatives (*p* = 0.00001), and the GIS website (*p* = 0.02)—the correlation with medical representatives is relatively weak, while the others are weak.

Doctors primarily sourced information from radio (*p* < 0.001), while television was mainly used by doctors and nurses/midwives (*p* < 0.001). Industry conferences (*p* < 0.001) and medical representatives (*p* < 0.001) were, primarily, sources for doctors and pharmacists/pharmacy technicians—the correlation with medical representatives is relatively weak, and the others are weak. Pharmacists significantly more often used professional articles (*p* < 0.001) and the GIS website (*p* = 0.01)—the correlation with professional articles is relatively weak, while the correlation with the GIS website is weak.

**Figure 2:**
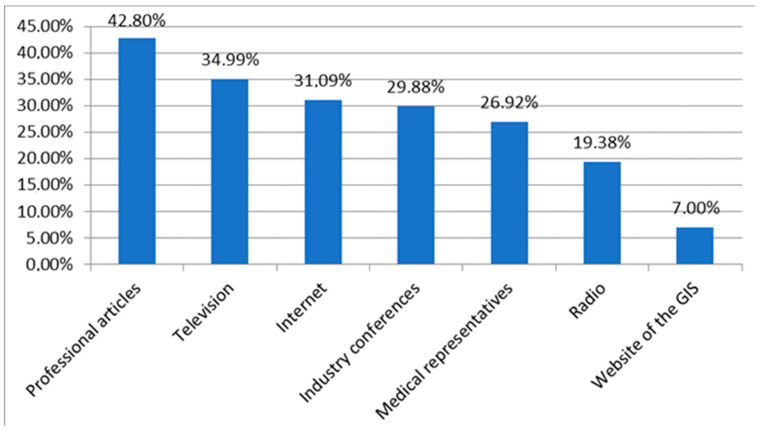
Sources of knowledge for respondents about dietary supplements. Multiple choice questions (GIS—Chief Sanitary Inspectorate).

Respondents with a specialist title were less likely to obtain knowledge from television (*p* < 0.001) and more likely to use industry conferences (*p* = 0.03), professional articles (*p* < 0.001), and the GIS website (*p* = 0.002) (Figure 2).

**Figure 3:**
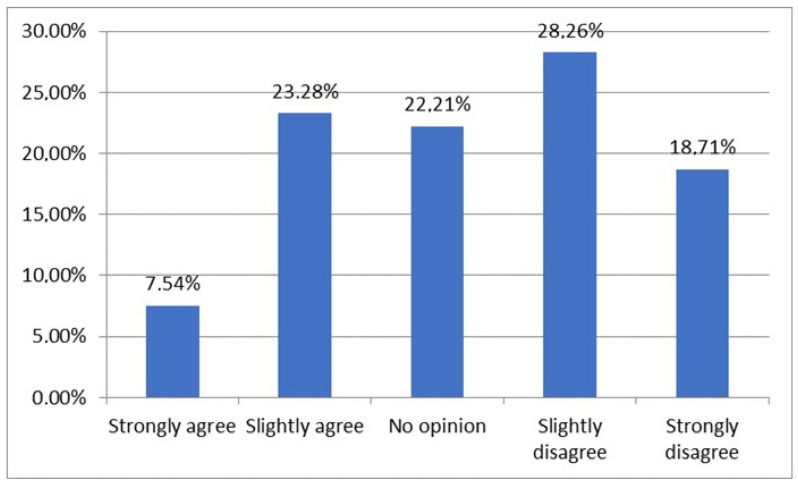
To what extent do you agree with the statement “I am influenced by advertising when choosing a dietary supplement”.

The majority of respondents (46.97%) stated that they do not consider advertising when choosing a dietary supplement, while nearly one in four (22.21%) were unable to express an opinion on this matter (Figure 3). Advertising appears to have a slightly greater influence on doctors (compared to other professional groups) (*p* < 0.001) and on those without a specialist title (*p* < 0.001).

Composition (84.52%), manufacturer (75.91%), and price (66.76%) influence the choice of a dietary supplement recommended by the majority of respondents, with composition having the most significant impact (as indicated by the highest percentage of “strongly agree” responses) (Table 2). Women (*p* < 0.001) significantly more often, and nurses/midwives significantly less often than other professions (*p* < 0.001), consider composition in their recommendations—both correlations are relatively weak. Women (*p* < 0.001) and nurses/midwives (*p* < 0.001) also significantly more often consider the manufacturer—both correlations are relatively weak. Women (*p* < 0.001), doctors (*p* < 0.001), and those without a specialist title are more likely to consider price—the correlation with women is weak, while the others are relatively weak.

The highest percentage of respondents, 22.21%, indicated that they recommend dietary supplements “once a week”. This is followed by 18.44% who recommend them “once every few weeks” and 17.63% who recommend them “almost daily”. Additionally, 15.88% of respondents recommend dietary supplements “several times a month”, while 15.21% reported that they “never” recommend dietary supplements. The smallest proportion, 10.63%, stated that they recommend dietary supplements “daily”.

This distribution suggests that a significant portion of healthcare professionals regularly recommends dietary supplements, with the majority doing so on a weekly basis, while a smaller percentage avoids recommending them entirely.

Daily recommendation is more common among women (*p* < 0.001) and less common among nurses/midwives (*p* < 0.001).

The proportion of respondents who agreed and disagreed with the statements “A dietary supplement is a medicine/medicinal product.” and “Dietary supplements can replace a balanced diet.” was similar, amounting to 42.67% vs. 39.03% and 38.23% vs. 45.09%, respectively.

For the other questions in Table 3, at least half of the respondents agreed with the statement being studied. However, the weakest agreement was observed with the last statement (69.32% vs. 65.28% vs. 51.54%).

“A dietary supplement is a medicine/medicinal product.”—Women (*p* < 0.001) and individuals without specialization (*p* = 0.01) were more likely to agree with this statement. Both relationships are weak.

“Dietary supplements can replace a balanced diet.”—Nurses/midwives (*p* < 0.001) and individuals without specialization (*p* = 0.008) were more likely to agree with this statement. The first relationship is rather weak, while the second is weak.

“Dietary supplements complement a normal diet.”—Women (*p* < 0.001) were more likely to agree with this statement, whereas nurses/midwives (*p* < 0.001) were less likely to agree.

“Dietary supplements are food products.”—Pharmacists/pharmacy technicians (*p* < 0.001) were less likely to agree with this statement, indicating a rather weak relationship.

“Dietary supplements have a nutritional effect.”—Doctors (*p* < 0.001) were more likely to agree with this statement, indicating a rather weak relationship.

More than half of the respondents agreed with each of the statements presented in Table 4, except for the second one (62.86% vs. 43.48% vs. 76.04% vs. 77.93% vs. 68.24%).

“Dietary supplements can have a negative impact on the body.”—Men (*p* < 0.001) and pharmacists/pharmacy technicians (*p* < 0.001) were more likely to agree with this statement. The first relationship is weak, while the second is rather weak.

“Dietary supplements are as safe as medicines.”—Women (*p* < 0.001) and individuals without specialization (*p* = 0.004) were more likely to agree with this statement, whereas pharmacists/pharmacy technicians (*p* < 0.001) were less likely to agree. The latter relationship is rather weak, while the others are weak.

“Dietary supplements with the same composition can vary in quality.”—Women (*p* < 0.001) and individuals with specialization (*p* < 0.001) were more likely to agree with this statement. The first relationship is weak, while the second is weak.

“Dietary supplements should be purchased from reliable sources.”—Women (*p* < 0.001) were more likely to agree with this statement, whereas nurses/midwives (*p* < 0.001) were less likely to agree. Both relationships are rather weak.

The vast majority of respondents agreed with the statement “Dietary supplements are subject to the Food Safety and Nutrition Act.” (72.14%), while slightly more than half agreed with the statement “The packaging of dietary supplements looks the same as medicine packaging.” (53.57%). For the remaining statements in Table 5, the percentage did not exceed 50% (41.72% vs. 49.26% vs. 45.35%).

“The packaging of dietary supplements looks the same as medicine packaging.”—Women (*p* < 0.001), doctors (*p* < 0.001), and individuals without specialization (*p* < 0.001) were more likely to agree with this statement. Each of these relationships is rather weak.

“Dietary supplements are subject to the Pharmaceutical Law.”—Nurses/midwives (*p* < 0.001) and individuals without specialization (*p* = 0.007) were more likely to agree with this statement. The first relationship is rather weak, while the second is weak.

“Dietary supplements are subject to the Food Safety and Nutrition Act.”—Women (*p* = 0.00000) and individuals without specialization (*p* < 0.001) were more likely to agree with this statement, whereas nurses/midwives (*p* < 0.001) were less likely to agree. Each of these relationships is rather weak.

“The procedure for marketing dietary supplements and medicinal products is the same.”—Men (*p* < 0.001), nurses/midwives (*p* < 0.001), and individuals without specialization (*p* < 0.001) were more likely to agree with this statement. Each of these relationships is rather weak.

“The packaging of dietary supplements includes a marketing authorization number.”—Pharmacists/pharmacy technicians were least likely to agree with this statement, while nurses/midwives (*p* < 0.001) were the most likely to agree. Individuals without specialization also agreed more frequently with this statement (*p* < 0.001). Each of these relationships is rather weak.

The vast majority of respondents agreed with each of the statements from Table 6 (especially the first one), except for the second statement (83.72% vs. 48.45% vs. 73.08% vs. 69.31%).

“The packaging of dietary supplements must include the term ‘dietary supplement.’”—Nurses/midwives (*p* < 0.001)were more likely to agree with this statement. This relationship is rather weak.

“I use the list of dietary supplements published on the website of the Chief Sanitary Inspectorate.”—Men (*p* = 0.002) and doctors were more likely to agree with this statement, while pharmacists/pharmacy technicians were least likely to agree (*p* < 0.001). The first relationship is weak, while the second is rather weak.

“Dietary supplements can interact with medications.”—Pharmacists/pharmacy technicians (*p* < 0.001) and individuals with specialization (*p* = 0.001) were more likely to agree with this statement. Each of these relationships is rather weak.

“Dietary supplements can affect the effectiveness of prescription medications.”—Women (*p* < 0.001) and pharmacists/pharmacy technicians (*p* < 0.001) were more likely to agree with this statement. Each of these relationships is rather weak.

Among the reasons for recommending dietary supplements, respondents primarily mentioned the need to address nutritional deficiencies and improve the body’s immunity (59.19%), pregnancy (18.38%), the patient’s experience of excessive fatigue (16.91%), and bone health issues (5.51%). When recommending dietary supplements, respondents primarily inquire about the medications/supplements being taken, coexisting conditions (52.02%), the consumer’s diet (38.15%), and allergies (9.83%). Those who decide to recommend dietary supplements mainly limit themselves to vitamins and macro-/microelements.

## 4. Discussion

The results of this study provide valuable insights into the knowledge, attitudes, and practices of healthcare professionals regarding dietary supplements. While dietary supplements are widely used, opinions about their role in health and well-being remain mixed. This discussion highlights the most significant findings and compares them with existing literature to offer a broader understanding of the study’s context.

A substantial majority of respondents agreed with the statement, “I understand and know what a dietary supplement is”, with 38.09% strongly agreeing and 35.40% slightly agreeing. Women, pharmacists, and specialists were more likely to declare familiarity with the term. These findings reflect a relatively high level of awareness among healthcare professionals, as supported by the literature, which shows growing awareness of dietary supplements in this group. However, it is important to recognize that not all professional groups possess the same depth of knowledge in this area. Other studies have shown that healthcare workers’ knowledge about dietary supplements, particularly in terms of composition and potential drug interactions, can sometimes be limited, even in other countries [11,12].

The most frequently cited source of knowledge about dietary supplements was specialist articles (42.80%), while only 7.00% of respondents used official sources such as the Chief Sanitary Inspectorate (GIS). This reliance on scientific literature and professional conferences is consistent with other research showing that healthcare workers often turn to professional sources for information [13,14,15]. The low interest in information from GIS highlights the need to better promote official resources among healthcare professionals, which could help improve their understanding of regulatory frameworks and the safety of supplements. The data also revealed variations in preferences based on gender and specialization, underscoring the importance of tailoring information campaigns to specific professional groups.

An interesting finding is that nearly half (46.97%) of the respondents reported not considering advertising when choosing dietary supplements, though 22.21% were neutral on this matter. Advertising appeared to have a slightly greater influence on doctors and non-specialists, indicating that some groups may be more susceptible to marketing. Similar patterns have been observed in the literature, where marketing influences decisions regarding supplements, particularly in groups with less established knowledge of product composition and effects [3,15]. Although these correlations are not strong, they point to the importance of fostering critical thinking about marketing information.

Composition (84.52%), manufacturer (75.91%), and price (66.76%) were the top factors influencing supplement choices among respondents. The emphasis on composition highlights healthcare workers’ concern with the quality and content of the supplements they recommend. Pharmacists and women were especially attentive to product composition, possibly due to their roles in daily consumer care and greater familiarity with supplement ingredients. The literature also emphasizes the significance of active ingredients and manufacturers in healthcare professionals’ decision-making processes [3,16].

Most respondents (22.21%) recommended dietary supplements at least once a week, with only 10.63% advising daily use. This suggests that healthcare professionals generally view supplements as occasional additions to a patient’s diet rather than as part of a routine regimen. Women were more likely to recommend daily supplementation, possibly due to their more frequent involvement in preventive care.

The study also revealed some uncertainty about the role of dietary supplements. Opinions were divided on whether “a dietary supplement is a drug/medicinal product” and whether “dietary supplements can replace a balanced diet”. Women and non-specialists were more likely to view supplements as drugs, suggesting that these groups may lack a full understanding of the differences between supplements and medicinal products. This finding aligns with other studies that highlight the need for better training, particularly for medical students and less experienced professionals, on the appropriate use of dietary supplements [17,18,19,20].

Finally, more than half of the respondents acknowledged that dietary supplements could have negative effects on the body. Women and pharmacists demonstrated greater awareness of these risks, further emphasizing the variability in knowledge across professional groups. The literature frequently highlights the potential dangers of dietary supplements, such as overdosing on vitamins or drug-supplement interactions, even though supplements are often perceived as “safe” [21,22,23,24]. These results underline the critical need for targeted educational initiatives to better inform healthcare professionals about the safe and effective use of dietary supplements. This is particularly important in view of the role of professionals as patient advisors. It should be noted that studies, including those conducted in Poland, indicate frequent use of dietary supplements among the population and beliefs about the effectiveness of using dietary supplements, especially those of natural origin [25]. Moreover, the literature indicates numerous interactions that can occur as a result of using herbs and dietary supplements alone or concomitantly with medications. Proper knowledge of the use of these products can prevent many of these types of situations [26].

This discussion shows that, while healthcare professionals demonstrate a broad understanding of dietary supplements, significant gaps in knowledge remain, particularly in relation to safety, composition, and regulation. Focused education and awareness efforts are essential to ensure that healthcare workers can provide accurate and responsible recommendations to patients.

### Limitations of the Study

Despite the valuable insights provided by this study, several limitations should be noted. The primary limitation is the lack of formal validation and standardization of the questionnaire used to assess healthcare professionals’ knowledge and practices regarding dietary supplements. While the questionnaire was developed based on relevant literature and underwent a pilot test with six healthcare workers from the target group (three pharmacists, two doctors, and one nurse), no significant changes were suggested. However, the absence of a formal validation process means that the tool’s reliability and accuracy in capturing the intended constructs remain uncertain.

Additionally, the sample shows an imbalance in the representation of different healthcare professions, with doctors making up 54.24%, pharmacists and pharmacy technicians 28.13%, and nurses 17.63%. This uneven distribution may influence the comparison of knowledge and practices across groups. Future studies should aim for a more balanced sample to ensure the findings are more representative of all healthcare professionals.

## 5. Conclusions

The study indicates significant differences in the knowledge of healthcare professionals regarding dietary supplements, which are correlated with factors such as gender, place of residence, and profession. These results emphasize the need for systematic supplementation of specialists’ knowledge about dietary supplements, which can lead to more informed and responsible counseling of patients. Moreover, up-to-date knowledge of healthcare professionals is crucial for raising general public awareness of dietary supplements. Education and access to reliable information can significantly influence consumers’ decisions regarding supplementation, which in turn will contribute to improving the quality of healthcare and consumer safety. Understanding and implementing these aspects in clinical practice is essential to ensure that recommendations regarding dietary supplements are consistent with the latest scientific evidence and tailored to individual consumer needs.

## Figures and Tables

**Table 1:** Demographic characteristics of the study group.

	Study Group (*n* = 743)
Age	
median (1st–3rd quartile)	44 (36–51)
range	22–70
Sex	
Female	400 (53.84%)
Male	343 (46.16%)
Job	
Doctor	403 (54.24%)
Pharmacist/pharmacy technician	209 (28.13%)
Nurse/midwife	131 (17.63%)
Years in the profession	
median (1st–3rd quartile)	15 (9–22)
range	1–53
Size of permanent residence:	
Village	44 (5.92%)
Town up to 10,000 inhabitants	56 (7.54%)
Town 10,000–50,000 inhabitants	107 (14.40%)
Town 50,000–100,000 inhabitants	118 (15.88%)
Town 100,000–500,000 inhabitants	127 (17.09%)
City above 500,000 inhabitants	291 (39.17%)

**Table 2:** Respondents’ answers to the question, “What factors do you consider when recommending a dietary supplement?”.

	What Factors Do You Consider When Recommending a Dietary Supplement? (*n* = 743)		
	Composition	Manufacturer	Price
Strongly disagree	5 (0.67%)	11 (1.48%)	39 (5.25%)
Slightly disagree	17 (2.29%)	57 (7.67%)	86 (11.57%)
No opinion	93 (12.52%)	111 (14.94%)	122 (16.42%)
Slightly agree	317 (42.66%)	356 (47.91%)	326 (43.88%)
Strongly agree	311 (41.86%)	208 (28.00%)	170 (22.88%)

**Table 3:** Respondents’ answers to questions regarding general knowledge about dietary supplements.

	To What Extent Do You Agree with the Statement… (*n* = 743)				
	A Dietary Supplement Is a Medicine/Medicinal Product.	Dietary Supplements Can Replace a Balanced Diet.	Dietary Supplements Complement a Normal Diet.	Dietary Supplements Are Food Products.	Dietary Supplements Have a Nutritional Effect.
Strongly disagree	184 (24.76%)	177 (23.82%)	20 (2.69%)	64 (8.61%)	57 (7.67%)
Slightly disagree	106 (14.27%)	158 (21.27%)	72 (9.69%)	56 (7.55%)	96 (12.92%)
No opinion	136 (18.30%)	124 (16.69%)	136 (18.30%)	138 (18.57%)	207 (27.86%)
Slightly agree	192 (25.85%)	160 (21.54%)	337 (45.36%)	335 (45.09%)	235 (31.62%)
Strongly agree	125 (16.82%)	124 (16.69%)	178 (23.96%)	150 (20.19%)	148 (19.92%)

**Table 4:** Respondents’ answers to questions about the safety of dietary supplements.

	To What Extent Do You Agree with the Statement… (*n* = 743)				
	Dietary Supplements Can Have a Negative Impact on the Body.	Dietary Supplements Are as Safe as Medicines.	Dietary Supplements with the Same Composition Can Vary in Quality.	Dietary Supplements Should be Purchased from Reliable Sources.	Dietary Supplements from Well-Known Manufacturers Have Better Quality.
Strongly disagree	35 (4.71%)	123 (16.55%)	5 (0.67%)	6 (0.81%)	20 (2.69%)
Slightly disagree	114 (15.34%)	122 (16.41%)	36 (4.85%)	24 (3.23%)	83 (11.17%)
No opinion	127 (17.09%)	175 (23.56%)	137 (18.44%)	134 (18.03%)	133 (17.91%)
Slightly agree	261 (35.13%)	204 (27.46%)	303 (40.78%)	202 (27.19%)	340 (45.76%)
Strongly agree	206 (27.73%)	119 (16.02%)	262 (35.26%)	377 (50.74%)	167 (22.48%)

**Table 5:** Respondents’ responses to questions about the regulation of dietary supplements.

	To What Extent Do You Agree with the Statement… (*n* = 743)				
	The Packaging of Dietary Supplements Looks the Same as Medicine Packaging.	Dietary Supplements Are Subject to the Pharmaceutical Law.	Dietary Supplements Are Subject to the Food Safety and Nutrition Act.	The Procedure for Marketing Dietary Supplements and Medicinal Products Is the Same.	The Packaging of Dietary Supplements Includes a Marketing Authorization Number.
Strongly disagree	98 (13.19%)	156 (21.00%)	0 (0.00%)	214 (28.80%)	133 (17.90%)
Slightly disagree	96 (12.92%)	137 (18.44%)	79 (10.63%)	75 (10.09%)	70 (9.42%)
No opinion	151 (20.32%)	140 (18.84%)	128 (17.23%)	88 (11.85%)	203 (27.32%)
Slightly agree	254 (34.19%)	199 (26.78%)	278 (37.42%)	220 (29.61%)	208 (27.99%)
Strongly agree	144 (19.38%)	111 (14.94%)	258 (34.72%)	146 (19.65%)	129 (17.36%)

**Table 6:** Respondents’ responses to questions about the safety of taking dietary supplements.

	To What Extent Do You Agree with the Statement… (*n* = 743)			
	The Packaging of Dietary Supplements Must Include the Term “Dietary Supplement.”	I Use the List of Dietary Supplements Published on the Website of the Chief Sanitary Inspectorate.	Dietary Supplements can Interact with Medications.	Dietary Supplements can Affect the Effectiveness of Prescription Medications.
Strongly disagree	0 (0.00%)	133 (17.90%)	6 (0.81%)	15 (2.02%)
Slightly disagree	37 (4.97%)	78 (10.50%)	78 (10.50%)	68 (9.15%)
No opinion	84 (11.31%)	172 (23.15%)	116 (15.61%)	145 (19.52%)
Slightly agree	315 (42.40%)	224 (30.15%)	248 (33.38%)	238 (32.03%)
Strongly agree	307 (41.32%)	136 (18.30%)	295 (39.70%)	277 (37.28%)

## Data Availability

All data are available from the corresponding author.
